# Independent Component Analysis of the Effect of L-dopa on fMRI of Language Processing

**DOI:** 10.1371/journal.pone.0011933

**Published:** 2010-08-17

**Authors:** Namhee Kim, Prem K. Goel, Madalina E. Tivarus, Ashleigh Hillier, David Q. Beversdorf

**Affiliations:** 1 Department of Statistics, The Ohio State University, Columbus, Ohio, United States of America; 2 Department of Imaging Science and the Rochester Center for Brain Imaging, University of Rochester, Rochester, New York, United States of America; 3 Department of Psychology, University of Massachusetts-Lowell, Lowell, Massachusetts, United States of America; 4 Departments of Radiology, Neurology and Psychology, University of Missouri, Columbia, Missouri, United States of America; 5 The Thompson Center, University of Missouri, Columbia, Missouri, United States of America; Cuban Neuroscience Center, Cuba

## Abstract

L-dopa, which is a precursor for dopamine, acts to amplify strong signals, and dampen weak signals as suggested by previous studies. The effect of L-dopa has been demonstrated in language studies, suggesting restriction of the semantic network. In this study, we aimed to examine the effect of L-dopa on language processing with fMRI using Independent Component Analysis (ICA). Two types of language tasks (phonological and semantic categorization tasks) were tested under two drug conditions (placebo and L-dopa) in 16 healthy subjects. Probabilistic ICA (PICA), part of FSL, was implemented to generate Independent Components (IC) for each subject for the four conditions and the ICs were classified into task-relevant source groups by a correlation threshold criterion. Our key findings include: (i) The highly task-relevant brain regions including the Left Inferior Frontal Gyrus (LIFG), Left Fusiform Gyrus (LFUS), Left Parietal lobe (LPAR) and Superior Temporal Gyrus (STG) were activated with both L-dopa and placebo for both tasks, and (ii) as compared to placebo, L-dopa was associated with increased activity in posterior regions, including the superior temporal area (BA 22), and decreased activity in the thalamus (pulvinar) and inferior frontal gyrus (BA 11/47) for both tasks. These results raise the possibility that L-dopa may exert an indirect effect on posterior regions mediated by the thalamus (pulvinar).

## Introduction

Cognitive tasks such as those involving language require the integration of a wide range of brain regions. Previous research [1]–[4] reveals that anterior Left Inferior Prefrontal Cortex (LIPC) (BA 45/47/10), posterior LIPC (BA 44/45/46) and posterior Left Middle Temporal Gyrus (LMTG) (BA21), bilateral fusiform gyrus, bilateral cerebellum, left dorsal caudate and ventral anterior thalamus are involved in a variety of language tasks. More specifically, the role of the left inferior prefrontal cortex appears to be implicated in the selection of competing words in semantic language tasks [3]. Both the posterior and anterior LIPC areas (BA 45/47) are typically activated during semantic tasks whereas the posterior LIPC (BA 44/45) area is preferentially activated in tasks which involve attending to phonology, involving decisions regarding auditory syllables or rhymes [1], [2]. However, such a spatial division in the LIPC for semantics and phonology was not significant when the two tasks are directly contrasted [5]. The left Middle Temporal Gyrus (MTG) (BA21) is known to be involved in auditory information processing. Similar temporal activation on fMRI is found for both semantic and phonological tasks [3], [4]. It is believed that the visually presented verbal stimuli invoke the sound of the word automatically via network activation, manifested by left MTG activation. Moreover, bilateral fusiform gyrus (BA37), bilateral cerebellum, left dorsal caudate and ventral anterior thalamus were found to be activated in both semantic and phonological tasks [4].

Dopaminergic neurons are present mainly in the ventral tegmental area (VTA) of the midbrain, substantia nigra, and the subthalamic nucleus. Dopaminergic projections arise from the VTA and then project diffusely across the frontal cortex [6], [7]. Dopamine has a major role in regulating motor function. The loss of dopamine neurons in the substantia nigra results in Parkinson disease, with a loss in the ability to initiate controlled movements. From a cognitive standpoint, the dopaminergic system has effects on cognitive flexibility of the set shifting type [8]–[10], reversal learning [11], spatial planning [12], [13], and working memory [14], [15], all of which are tasks highly dependent on frontal lobe function. Dopamine also appears to modulate a signal-to-noise ratio, strengthening strong signals and dampening weak signals [16]. According to this model, decreased dopaminergic activation of cortical areas leads to a decrease in the functional focus of cortical neuronal network activity, whereas increased dopaminergic activation leads a high signal-to-noise ratio. However, it should be noted that the effects of dopaminergic treatment, as evidenced by studies on cognition in Parkinson's disease as well as studies in animal models, are characterized by an inverted “U”-shaped response curve, such that increasing doses of dopaminergic agonists can improve or deteriorate performance on executive function-related tasks [17].

The administration of L-dihydroxypheylalanine (L-dopa), the precursor of the dopamine, has been found to cause restriction of the semantic network in a semantic priming task [16]. Subjects were presented with a series of word pairs in which the first word was paired with a closely related word, a distantly related word, a non-related word, or a non-word. Subjects then performed a lexical decision task in which they were asked to decide whether the second series of letters was a word or a non-word. In the placebo condition, subjects recognized the closely and distantly related words more quickly than non-related words. L-dopa treatment resulted in significantly quicker recognition of closely related words than non-related words, but the recognition latencies of distantly related words and non-related words were not significantly different, providing evidence of the restriction of the semantic network.

Dopaminergic modulation of the semantic network using functional Magnetic Resonance Image (fMRI) was examined by Tivarus et al. [18]. The analysis revealed a change in functional connectivity (FC) in one pair of brain regions, left fusiform gyrus (LFUS) and left parietal lobe (LPAR) with L-dopa. However, as dopamine projects predominantly to the frontal lobe [6], [7], it was unclear why only these more posterior regions were affected. Although, as these posterior brain regions (LFUS and LPAR) are important for word recognition, such an effect on FC might otherwise be expected due to the aforementioned effects of L-dopa on priming [16].

We wished to determine whether other brain regions, undetected in previous work using general linear model (GLM) [18], might act as an intermediary between the frontal targets of dopaminergic projections and the posterior brain regions which demonstrate the FC effect of L-dopa. Therefore, in this study, we use the Independent Component Analysis (ICA), a nonparametric analysis tool for independent source separation, on fMRI data to examine the differences in language processing between L-dopa and placebo groups. The underlying experimental paradigm is not designed for detection of behavioral effects, as it is a simple task where the performance is at ceiling [18]. Rather, the purpose is to examine whether ICA reveals novel information regarding how frontal dopaminergic projections may affect the more posterior language areas as compared to placebo.

## Results

**Figure 1:**
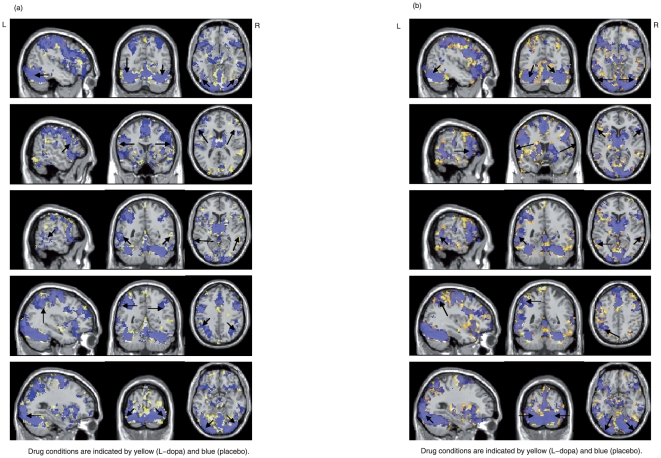
Group activation map. (a) The activation map with the phonological task was obtained by FDR of 5% and spatial extent significance level of 5% which corresponds to 40 voxels. Drug conditions are indicated by yellow (L-dopa) and blue (placebo). From top, three-dimensional representations of bilateral fusiform gyrus (BA37), bilateral inferior frontal gyrus (BA44/45), bilateral posterior superior temporal gyrus (BA22), bilateral inferior parietal lobe (BA40) and bilateral occipital gyrus (BA19) are presented; (b) The activation map with the semantic task was obtained by FDR of 5% and spatial extent significance level of 5% which corresponds to 40 voxels. Drug conditions are indicated by yellow (L-dopa) and blue (placebo). From top, three-dimensional representations of bilateral fusiform gyrus (BA37), bilateral inferior frontal gyrus (BA44/45), bilateral posterior superior temporal gyrus (BA22), left inferior parietal lobe (BA40) and bilateral occipital gyrus (BA19) are presented.

**Figure 2:**
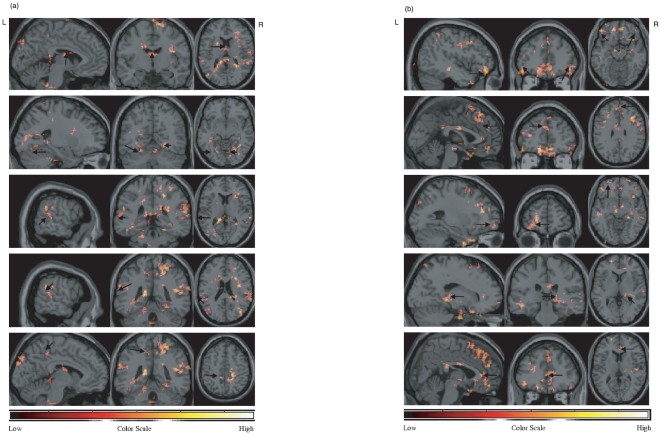
Differentially activated regions by drug conditions in the phonological task. (a) The regions showing greater activation with L-dopa were obtained by voxel level significance of 5% (uncorrected) and spatial extent significance level of 5% (uncorrected) which corresponds to 70 voxels. From top, three-dimensional representations of mediodorsal thalamus, bilateral fusiform gyrus (BA 37), left posterior temporal gyrus (BA 22), inferior parietal lobe near supramarginal gyrus (BA 40), and posterior cingulate gyrus (BA 31) are presented. (b) The regions showing greater activation with placebo were obtained by voxel level significance of 5% (uncorrected) and spatial extent significance level of 5% (uncorrected) which corresponds to 70 voxels. From top, three-dimensional representations of bilateral inferior frontal regions (BA 11/47), medial frontal gyrus (BA 9), superior frontal gyrus (BA 10), pulvinar, and anterior cingulate gyrus (BA 24) are presented.

**Figure 3:**
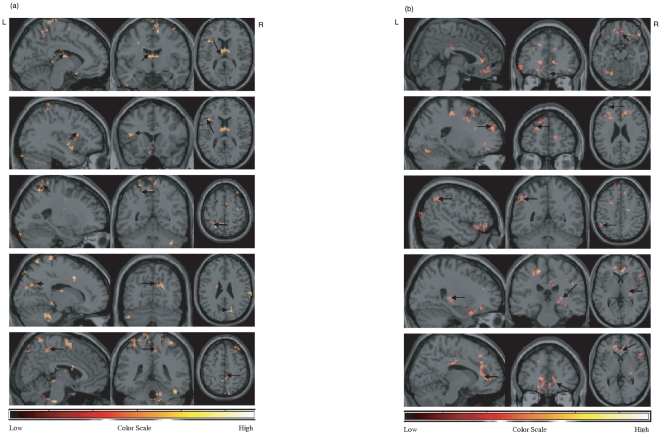
Differentially activated regions by drug conditions in the semantic task. (a) The regions showing greater activation with L-dopa were obtained by voxel level significance of 5% (uncorrected) and spatial extent significance level of 5% (uncorrected) which corresponds to 70 voxels. From top, three-dimensional representations of mediodorsal thalamus, left insula, inferior parietal lobe (BA 40), cuneus (BA 18), and posterior cingulate gyrus (BA 31) are presented. (b) The regions showing greater activation with placebo were obtained by voxel level significance of 5% (uncorrected) and spatial extent significance level of 5% (uncorrected) which corresponds to 70 voxels. From top, three-dimensional representations of inferior frontal regions (BA 11/47), medial frontal gyrus (BA 9), inferior parietal lobe (BA 40), pulvinar, and anterior cingulate gyrus (BA 24) are presented.

No side effects were observed with drug administration in this study [18]. There are four experimental conditions derived from the combination of two drug types (L-dopa and placebo) and two task types (phonological and semantic). The BOLD signals for each subject were analyzed using ICA and post-processed, which led to an individual summary map (see Materials and Methods for details). This collection of summary maps was used as input to the flexible factorial model of SPM5. The group activation maps produced by SPM(t) for the phonological and the semantic tasks are presented in Figure 1a and 1b respectively, in which activation with placebo and L-dopa is indicated by different colors. Since our primary focus was to examine the difference in activation between drugs (L-dopa, placebo), the two drug conditions (L-dopa, placebo) were tested under each task condition. The results of this analysis are shown in Figure 2 and 3. McDermott et al. [4] and Tivarus et al. [18] used GLM to test the difference between activations under the two task conditions with placebo. We repeat this comparison in order to compare the results based on our method with those based on GLM.

### Group activation maps

Regions activated by both tasks during both drug conditions are bilateral inferior frontal cortex (BA 44/45/47) which extends through premotor and motor areas (BA 4/6), bilateral cerebellum, bilateral occipital cortex (BA 17/18/19), bilateral fusiform gyrus (BA37), bilateral posterior superior and middle temporal gyrus (BA 21/22), thalamus, bilateral superior and middle frontal gyrus (BA 9/46) and left parietal lobe (bilateral for phonological task) (BA40). The results are presented in Figure 1a and 1b, where activated regions with placebo and L-dopa are indicated in blue and yellow, respectively.

### Differentially activated regions between drug conditions during the phonological task

The regions preferentially activated with L-dopa were left cerebellum, bilateral occipital cortex, bilateral posterior superior temporal gyrus (BA 22), bilateral fusiform gyrus (BA 37), thalamus (mediodorsal), posterior cingulate (BA 31), and bilateral inferior parietal lobe (BA 40) near the supramarginal gyrus whereas the regions preferentially activated with placebo were bilateral superior, middle and inferior frontal gyrus (BA 9/10/11/47), anterior cingulate (BA 24/32), thalamus (pulvinar), and bilateral inferior parietal gyrus (BA 40) near the supramarginal gyrus which are located superior to the region activated with L-dopa. The results are presented in Figure 2a and 2b.

### Differentially activated regions between drug conditions during the semantic task

The regions preferentially activated with L-dopa were right cerebellum (medial), thalamus (mediodorsal), left insula, cuneus (BA 18), inferior parietal lobe (BA 40) and posterior cingulate (BA 31) whereas the regions preferentially activated with placebo were left middle and superior frontal gyrus extending to the medial frontal lobe (BA 46/9), bilateral inferior frontal gyrus (BA 11/47), anterior cingulate gyrus (BA 24/32), thalamus (pulvinar), and bilateral inferior parietal lobe (BA 40) near the supramarginal gyrus. The results are presented in Figure 3a and 3b.

### Functional connectivity of thalamic regions of interest

Since dopamine projects heavily to frontal areas, but posterior areas were revealed to be affected by L-dopa in this and Tivarus et al. [18] studies, and thalamic areas were also affected by L-dopa in this study, we wished to examine post hoc the functional connectivity of the thalamic areas with frontal and posterior areas to examine support for the hypothesis that effects on posterior regions might be mediated by indirect effects from frontal dopaminergic projections by actions on the thalamus.

The pulvinar and the medial dorsal thalamus were revealed to have significant time series correlation, as our measure of connectivity, with BA 9, 11, and 47 among frontal regions and BA 20, 21, 22, 37 and 40 among posterior regions. Among these ROI pairs, significant increases in connectivity were observed with L-dopa for the pulvinar-BA11 and mediodorsal thalamus-BA11 ROI pairs, particularly for the semantic task. Increases with L-dopa were also observed for posterior connections as well for the semantic task, as observed for pulvinar-BA37, and mediodorsal thalamus-BA20. Increases in connectivity with L-dopa are also observed in some thalamic connections for the phonological task as well (mediodorsal thalamus-BA9, mediodorsal thalamus-BA21, with a decrease found between both thalamic regions and BA20 with L-dopa). The correlations are presented in Table 1.

**Table 1:** Correlations between thalamic and cortical regions.

		Semantic	Task	Condition	Phonological	Task	Condition
	BA	Placebo	L-dopa	Difference	Placebo	L-dopa	Difference
Pulvinar	9	0.57***	0.55***	0.02	0.43***	0.46***	−0.03
	11	0.11	0.21*	−0.10*	0.25**	0.30**	−0.05
	47	0.40***	0.39***	0.01	0.26**	0.33***	−0.07
	20	0.28**	0.32**	−0.04	0.42***	0.34***	0.08*
	21	0.34***	0.34***	0.00	0.41***	0.46***	−0.05
	22	0.35***	0.39***	−0.04	0.52***	0.45***	0.07
	37	0.33***	0.46***	−0.13***	0.52***	0.48***	0.04
	40	0.46***	0.46***	0.00	0.52***	0.48***	0.04
MD thalamus	9	0.71***	0.68***	0.03	0.4***	0.52***	−0.12**
	11	0.26**	0.39***	−0.13***	0.18*	0.20*	−0.02
	47	0.39***	0.43***	−0.04	0.39***	0.29**	0.10*
	20	0.25**	0.38***	−0.13***	0.39***	0.23*	0.16***
	21	0.23*	0.20*	0.03	0.36***	0.47***	−0.11**
	22	0.41***	0.42***	−0.01	0.33***	0.30**	0.03
	37	0.52***	0.46***	0.06	0.43***	0.46***	−0.03
	40	0.43***	0.50***	−0.07	0.46***	0.48***	−0.02

*** p-value (uncorrected) 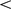 0.001, ** p-value 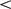 0.01, * p-value 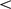 0.05.

### Differentially activated regions between tasks with placebo

The regions preferentially activated with the sematic task were left inferior frontal gyrus (BA 11/44/45/47), middle/medial frontal gyrus (BA 8/9/10), right inferior frontal gyrus (BA 44/45), left posterior superior/middle temporal gyrus (BA 21/22), bilateral superior temporal gyrus (BA38), bilateral cerebellum, left fusiform gyrus (BA37), posterior cingulate gyrus (BA23), supramarginal gyrus (BA40), and caudate head. These regions include the regions found by McDermott et al. [4] and Tivarus et al. [18] in the same condition.

The regions preferentially activated with the phonological task were left precentral gyrus (BA6), posterior inferior and anterior superior insula, bilateral inferior parietal lobe (BA40), left middle occipital gyrus (BA19), and anterior cingulate gyrus (BA32). Except for the anterior cingulate gyrus (BA 32), these results are also similar to previous studies by McDermott et al. [4] and Tivarus et al. [18].

## Discussion

In this study, a method for summarizing task-related ICs within subject for each task and drug condition is proposed. Selecting the independent component that contributes the most to a voxel, a higher sensitivity for the detection of differential activation seems to have been achieved. It should be noted that significant differences in activation maps between L-dopa and placebo were not found in GLM analysis [18]. The activated regions for both tasks include the anterior and posterior inferior prefrontal regions (BA 9/11/44/45/46/47) involved in the selection of competing words and the decision regarding auditory syllables or rhymes, fusiform gyrus (BA 37) involved in visual word form processing, the inferior parietal lobe and the superior and middle temporal gyrus (BA 21/22/40) involved in retrieval of word meaning [4], [19]–[22] or sound [4], [22] as expected with these language tasks. The cerebellum involved in motor control and language processing [23], motor (BA 6) and premotor (BA 4) involved in the motor task of button pressing, and occipital cortex (BA 17/18/19) involved in the visual stimuli processing were also activated for both tasks as expected. These activation maps are similar to the expected activation maps given the stimulus and response and the expected language activation for the phonological and the semantic tasks as per McDermott et al. [4] and Tivarus et al. [18].

In the comparison between drug conditions, effects of L-dopa on frontal regions may be expected due to the high degree of dopaminergic projection to the frontal lobes. However, posterior superior temporal gyrus (BA 22) was activated more with L-dopa than with placebo, as were the posterior cingulate (BA 31), bilateral fusiform gyrus (BA 37), and left inferior parietal lobe (BA 40). Many of these posterior regions of the brain are involved in retrieval of stored information: word meaning (posterior region of BA 21/22) [4], [19]–[22] or sound (BA 7/40) [4], [22]. Further, the posterior fusiform gyrus (BA 37) is involved with visual word form recognition [24]. Anatomically, the inferior parietal lobe (BA 40) lies in the region bounded ventrally by the superior and middle temporal gyrus (BA 21/22) and is a part of Wernicke's area, a region important in the speech comprehension. Therefore, since these posterior regions preferentially activated with L-dopa in our study, they seem to have importance in the effect of L-dopa on language processing. This suggests the possibility that L-dopa may affect a semantic network search to retrieve stored information through effects on these posterior areas of the brain. The increased activation in the left fusiform gyrus (BA 37) and left inferior parietal lobe (BA 40) with L-dopa may also relate to the findings by Tivarus et al. [18] in which the functional connectivity between left fusiform gyrus (BA 37) and left inferior parietal lobe (BA 40) was increased with L-dopa. This may suggest greater integration between these two regions and less outside integration of other inputs with drug, as might be expected with restricted access to the semantic network with L-dopa. In our study, the greater activation in fusiform gyrus and left inferior parietal lobe with L-dopa in the phonological task may relate to the spread of the activation from the visual word form processing area to the regions involved in the retrieving of the word's sound. These posterior effects, though, seem surprising given the distribution of dopamine projections which arise from ventral tegmental areas and spread primarily to the frontal cortex and much less to posterior regions [6], [7].

In the thalamus, anterior and dorsal medial regions showed greater activation with L-dopa whereas the pulvinar showed greater activation with placebo. The pulvinar region of the thalamus is known to project to posterior parietal lobe and inferior temporal gyrus [25]–[27] as well as the frontal cortex [26]–[28], and receive projections from the frontal and parietal cortices [29], whereas the dorsal medial thalamus projects to dorsal and medial prefrontal cortex [25], [30], and is known to be important for memory retrieval. Therefore, these posterior effects of L-dopa on activation detected in our study, as well as the functional connectivity effects previously reported on posterior areas [18], may be mediated indirectly by effects on pulvinar, where the decreased activity of the inhibitory GABAergic neurons in the pulvinar in the L-dopa condition might result in increased activity of its projections to the posterior parietal and inferior temporal areas. This is supported by the high degree of functional connectivity between these thalamic regions and the proposed associated frontal and posterior regions in this study in our post-hoc analysis, and the increased frontal-thalamic connectivity for many of these cortical areas with L-dopa during the semantic task, and for some regions the phonological task as well. The pulvinar is involved in visual processing and appears to participate in the cortical alarm system for subliminal fear [31]. However, the pulvinar has also been proposed to be involved with interacting with frontal and parietal areas during ambiguity resolution in language [32] and visual attention [33], which appears to be consistent with this hypothesis of the pulvinar mediating the frontal L-dopa effects on posterior regions. However, further research will be necessary to explore this possibility, as our data cannot address issues such as the pharmacology of these potential ROI interactions.

One drawback of the GLM analysis in fMRI reported previously [18], is that it assumes a linear association between each hemodynamic response function (HRF) and the expected task related HRF, but with noisy signal, it may not be able to detect significant differences. ICA, an independent source separation tool, is able to select only task relevant independent sources by using the proposed correlation threshold, which is expected to reduce noise and enhance sensitivity, yielding novel significant findings described herein. The second drawback of the earlier GLM analysis seems to be that it cannot represent the association between two lagged-time courses due to delayed response, since delayed response implies lack of linear association between two time courses. Furthermore, an activation map containing only the voxels that cross a strict voxelwise threshold based on temporal consistency with the task related HRF, may not be able to uncover the interconnected voxels with delayed and bidirectional relationships. Since ICA extracts spatial patterns of interest identified by applying a correlation threshold for each IC, it may possibly avoid rigid voxelwise thresholding and bring functionally connected voxels into the activation map. In this study, we aimed to use this technique to understand how these frontal dopaminergic projections may affect the more posterior language areas. As a partial answer, we found that this effect may be mediated indirectly by effects on the thalamus.

## Materials and Methods

fMRI data for each subject and for each session were collected with a 1.5 T General Electric (Milwaukee, WI) Signa scanner with a quadrature head coil. The BOLD contrast functional data were collected using a gradient echo EPI pulse sequence (TR = 3s; TE = 40ms; 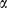 = 90; FOV = 240mm; matrix 64×64, 28 axial slices for whole brain coverage; 5mm slice thickness).

The two types of language tasks used in this experiment, semantic and phonological, were derived from McDermott et al. [4]. Tivarus et al. [18] used these tasks under two separate drug conditions, L-dopa and placebo. The fMRI data for the present study was obtained from the experiment by Tivarus et al. [18]. Sixteen right handed, native English speaking subjects (eight male and eight female, mean age 28.3 years with range of 21–49) without a history of psychiatric or neurological disease or learning disabilities (such as dyslexia) participated in this study. All subjects had normal or corrected-to-normal vision, and abstained from caffeine prior to the study to avoid hemodynamic effects from this agent. All subjects gave written consent in accordance with the Institutional Review Board of The Ohio State University, who specifically approved this study. The sample size in the proposed within-subjects analysis were expected to yield a significant L-dopa effect as suggested by previous research which yielded significant behavioral results [16] as well as significant imaging results [18], for L-dopa as compared to placebo with this sample size. The dose of L-dopa administered orally was 100mg, with 25mg carbidopa to block systemic effects, and testing was performed 90 minutes after administration to allow a peak level to be reached, as described in the previous research [18]. Drug was administered in a placebo-controlled, double blinded manner. Each participant received both drugs (L-dopa and placebo), one at each session. The two sessions were separated by at least one week. The order of drug administration was counterbalanced. During each test session, representing one drug condition, each participant performed two scanning runs. One of the runs consisted of alternating blocks of the semantic task (24 sec) and rest (30 sec), and the other run consisted of alternating blocks of the phonological task (24 sec) and rest (30 sec), for a total of 4 min and 6 seconds for each run. Each task block (24 sec) consisted of 15 words. In the semantic condition, 10 of the words in the list were related by meaning and 5 were unrelated to a cue word, while in the phonological condition 10 of the words rhymed and 5 did not rhyme with the cue word. The block design was as followed: a cue word was presented in capital letters for 3 seconds followed by the list of 15 words. Each word in the list was presented for approximately 1100ms, with a 300ms inter-stimulus interval (a blank screen), for a total of for 1.4 seconds for each word. Participants were instructed to attend to the meaning or sound of the lists presented. They responded by pressing one of two buttons YES or NO to indicate whether the word in the list was related to the cue by meaning or sound. Schematic diagrams of the two task conditions are described in detail in Figure S1 in the supporting information section.

BOLD signal is an indirect measure of neuronal activity and is thus affected by many other sources, including head movements during the scanning process, heart beat and respiratory related physiological changes in addition to the assigned cognitive task. Such sources contribute to the level of BOLD signal intensity in the functional MR image. A BOLD signal can be assumed to be a linear mixture of the sources influencing BOLD intensity level. Such sources may not be explained easily with parametric functions because of the presence of complex regional interactions within the brain and variations in activation across subjects. Independent Component Analysis has been used in Blind Source Separation (BSS), which does not need any parametric assumption but depends only on data for estimation of the independent sources. This analysis has been compared to a cocktail party problem, where a listener must separate the independent voices chattering at a cocktail party. ICA has been used in several fMRI studies [34]–[39]. ICA analysis denotes the observed BOLD signals for a subject during a given task and drug condition as a matrix X with T (the number of image acquisitions) rows and N (the number of voxels) columns. With linear ICA, X is modeled as a linear mixture of independent sources, i.e., 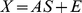, where A is a mixing matrix with T rows and Q (the number of sources) columns; S is a source matrix with Q rows and N columns; and E is a matrix of random errors. ICA searches for linear projections providing maximum independence of estimated sources without relying on any parametric assumptions, whereas GLM searches for the best linear projection based on the experimental design. Frequently used ICA algorithms to solve the aforementioned linear equation are the Infomax algorithm by Bell and Sejnowski [34] and the Fast ICA algorithm by Hyvärinen [40]. The Infomax algorithm maximizes the information transferred from inputs through a non-linear neural network transfer function whereas the Fast ICA algorithm maximizes independence among estimated ICs, which is achieved via maximization of negentropy, a measure of deviation from Gaussian distributions.

In this study, the Probabilistic Independent Component Analysis (PICA) algorithm proposed by Beckmann and Smith [37] was employed, which estimates sources by maximizing non-Gaussianity in terms of negentropy. In this estimation, spatial distribution of each source is assumed to have zero mean and unit variance. Since PICA assumes the presence of true signal as well as noise in the data, it is able to prevent over-fitting to noisy data. The number of sources for the decomposition for each subject was estimated using Laplace approximation to the posterior distribution of the model order and its mode [41]. The algorithm was implemented by utilizing Multivariate Exploratory Linear Optimized Decomposition into Independent Components (MELODIC), part of FSL, and the number of sources was generated for each subject in each experimental condition.

Since ICA is blind to any parametric assumption with regard to expected spatial and/or temporal patterns, it generates a number of independent sources irrespective of whether the estimated sources have any meaningful interpretation. Therefore, ICA requires a post-processing step to choose task-related ICs, which has been implemented by establishing thresholds on correlation between each IC's time course and the task-related hemodynamic response function (HRF), as explained in Text S1 in the supporting information section. Furthermore, as mentioned by Calhoun et al. [36], single-subject ICAs cannot be directly used to make a group inference. Various approaches for making an inference on a group of subjects with ICA have been proposed [36], [42], [43]. Schmithorst and Holland [43] described the methods of *subject-wise concatenation*, *row-wise concatenation*, and *across-subject averaging*. The subject-wise concatenation method [36] involves three steps, namely: (1) Principal Component Analysis (PCA) for each subject for dimension reduction, (2) Combine the Principal Components (PC) from each subject by concatenation for across-subject analysis using PCA, (3) ICA on the PCs from Step 2. On the other hand, the row-wise concatenation method [42] simply uses row-wise concatenated BOLD data (a kTxN matrix) in the ICA on the combined BOLD data across subjects. Finally, the across-subject averaging method [43] simply uses BOLD data averaged across subjects for the ICA. Based on a simulation, Schmithorst and Holland [43] concluded that subject-wise concatenation has better accuracy of estimated sources than the other two methods, when the number of subjects with simulated common components is relatively small and subject specific unique sources are present. It should be noted that these methods first obtain group level ICs based on combined data from all subjects, and then find subject-wise IC maps from the group level ICs. Thus the individual IC maps are based on all subjects. However, because of subject-to-subject variability in cognitive tasks, it may be better to estimate sources for each subject independently, irrespective of other subjects. Our approach for estimation of a unified task-related activation map based on one or more task-related ICs for a subject in a fixed task and drug condition, called the individual summary map, is described below. This collection of summary maps is then used as input to the SPM 5 for between- subject analysis.

**Figure 4:**
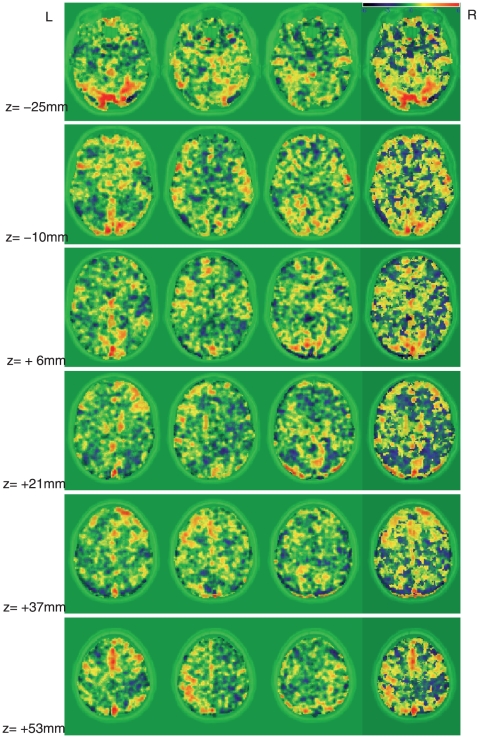
Task-related ICs and the summary map. Task-related ICs and the summary map at axial planes z = −25,−10, 6, 21, 37, 53 (mm) for Subject 8 with placebo during the phonological task. The first three images in each row are the task-related IC maps whereas the fourth image is the summary map made by the proposed method. Three IC maps are displayed in the order of the 2th, 7th and 18th ICs.

In linear ICA, BOLD response at location v in the brain for a subject is written as a linear combination of Q-independent sources 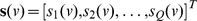 with time-varying mixing rates of each source. Calhoun et al. [36] interpret 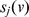 as the contribution (or weight) of the j-th source at location v. Out of the Q sources, we select only task-positive ICs based on thresholds for correlation between the time course of each source and the task-related HRF, which are established in post-IC processing. The ICs with time courses which showed a correlation beyond the threshold were selected as task-related. The correlation threshold was obtained by applying multiplicity correction with an independence assumption. Since the number Q of ICs generated by PICA differs for each subject for each condition, the correlation threshold applied for choosing task-related ICs differs for each subject, for each condition. (See Text S1 in the supporting information section for the details of the calculation). Furthermore, the number of ICs that met the correlation threshold criteria for each subject, for each task and drug condition was also different. The number of task-related ICs, 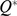, for each subject, for each task and drug condition is presented in Table 2. For example, for Subject 8, with placebo during the phonological task, three task-related ICs (IC #2, #7, and #18) were selected, and are displayed in the first three images of Figure 4. Whenever a subject showed multiple task-related ICs for a condition, another step was applied to summarize these sources in order to obtain the individual summary map. We compare absolute values of the contributions, 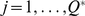 of task-related ICs at voxel v and assign the source value corresponding to the absolute maximum contribution to each subject's summary map. This rule can be described as follows.
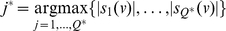
(1)

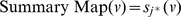
(2)


**Table 2:** Number of task-related ICs of subjects by task and drug conditions.

Subject	Phonological	task	Semantic	task
	L-dopa	Placebo	L-dopa	Placebo
1	1	2	1	1
2	1	1	3	1
3	5	1	3	3
4	1	2	2	3
5	4	4	2	2
6	2	2	4	1
7	1	2	1	2
8	4	3	1	4
9	3	5	3	3
10	2	2	3	1
11	2	2	1	1
12	3	3	2	3
13	3	2	4	1
14	1	1	3	1
15	1	3	2	3
16	1	3	2	1
Average	2.2	2.4	2.3	1.9

Since subject 7 in the semantic task with L-dopa has no ICs above the correlation threshold, an IC showing the greatest correlation to the task HRF was selected for the further analysis.

The fourth image of Figure 4 displays the summary map of Subject 8 for the phonological task with placebo. The above step provides each subject's summary map for each task and drug condition. For across-subjects analysis, Calhoun et al. [36] tested for voxel-wise significance on reconstructed individual ICs from an IC of Step 3 described above via one-sample t-test. In our study, we employed the same strategy to quantify statistical significance of the collection of individual summary maps.

The collection of individual summary maps, for each task and drug condition were then analyzed, voxel-by-voxel, via the flexible factorial model in SPM5 (http://www.fil.ion.ucl.ac.uk/spm) run in Matlab. The design matrix for the factorial model, as well as justification of the assumptions for the voxel-wise significance test are given in Text S2 in the supporting information section.

### Data Analysis

#### Preprocessing steps

Preprocessing preceded the described ICA. The preprocessing steps applied for this study were motion correction, spatial normalization to standard template and spatial smoothing as implemented in SPM5. The images were first corrected for head motion during the scan. The registered images were then normalized into the standard space provided by Montreal Neurological Institute (MNI) and smoothed using the Gaussian kernel with Full-Width Half-Maximum (FWHM) of 3mm×3mm×3mm.

#### Activation maps for experimental conditions

The summary maps for the conditions of task and drug for all subjects were then analyzed by 2 (tasks)×2 (drugs) flexible factorial model. The activation in each condition was determined by a False Discovery Rate (FDR) of 5% and by a spatial extent threshold of 5% (uncorrected). For the comparison between L-dopa and placebo for each task, and the comparison between the semantic task and the phonological task for placebo condition, the activation was determined by a voxel level threshold of 5% significance (uncorrected) and by a spatial extent threshold of 5% (uncorrected).

## Supporting Information

Figure S1 Design of the experiment and lists of words for the two tasks. The experimental design and an example of a list of words for each task are illustrated in Figure S1. Two separate block design experiments for two task conditions were employed at each drug session. In the list of words for the phonological task, “bottle”, “car”, “picture”, “pool”, and “horse” are not related with the cue word “deer” by rhyme or sound. In the list of words for the semantic task, “wheel”, “mouse”, “book”, “draw”, and “chair” are not related with the cue word “cold” by meaning.
(0.01 MB TIF)

Text S1 Correlation threshold calculation.
(0.05 MB PDF)

Text S2 Assumptions for the test statistics in the fexible factorial model.
(0.11 MB PDF)

## References

[1] Demonet J, Chollet F, Ramsay S, Cardebat D, Nespoulous J (1992). The anatomy of phonological and semantic processing in normal subjects.. Brain.

[2] Zatorre R, Evans A, Meyer E, Gjedde A (1992). Lateralization of phonetic and pitch discrimination in speech processing.. Science.

[3] Thompson-Schill S, D'Esposito M, Aguirre G, Farah M (1997). Role of left inferior prefrontal cortex in retrieval of semantic knowledge: a reevaluation.. Proc Natl Acad Sci USA.

[4] McDermott K, Pertersen S, Watson J, Ojemann J (2003). A procedure for identifying regions preferentially activated by attention to the semantic and phonological relations using functional magnetic resonance imaging.. Neuropsychologia.

[5] Devlin J, Matthews P, Rushworth M (2003). Semantic processing in the left inferior prefrontal cortex: a combined functional magnetic resonance imaging and transcranial magnetic stimulation study.. J Cogn Neurosci.

[6] Hall H, Sedvall G, Magnusson O, Kopp J, Halldin C (1994). Distribution of d1- and d2-dopamine receptors, and dopamine and its metabolites in the human brain.. Neuropsychopharmacol.

[7] Lidow M, Goldman-Rakic P, Gallager D, Rakic P (1991). Distribution of dopaminergic receptors in the primate cerebral cortex: Quantitative autoradiographic analysis using [^3^H]raclopride, [^3^H]spiperone and [^3^H]sch23390.. Neuroscience.

[8] Floresco S, Magyar O, Ghods-Sharifi S, Vexelman C, Tse M (2005). Multiple dopamine receptor subtypes in the medial prefrontal cortex of the rat regulate set-shifting.. Neuropsychopharmacology.

[9] Mehta M, Manes F, Magnolfi G, Sahakian B, Robbins T (2004). Impaired set-shifting and dissociable effects on tests of spatial working memory following the dopamine d2 receptor antagonist sulpiride in human volunteers.. Psychopharmacology.

[10] Cohen J, Braver T, Brown J (2002). Computational perspectives on dopamine function in prefrontal cortex.. Curr Opin Neurobiol.

[11] Mehta M, Swainson R, Oqilvie A, Sahakian J, Robbins T (2001). Improved short-term spatial memory but impaired reversal learning following the d2 agonist bromocriptine in human volunteers.. Psychopharmacology.

[12] Dagher A, Owen A, Boecker H, Brooks D (2001). The role of the striatum and hippocampus in planning: a pet activation study in parkinson's disease.. Brain.

[13] Rektorova I, Srovnalova H, Kubikova R, Prasek J (2008). Striatal dopamine transporter imaging correlates with depressive symptoms and tower of london task performance in parkinson's disease.. Mov Disorders.

[14] Winterer G, Weinberger D (2004). Genes, dopamine and cortical signal-to-noise ratio in schizophrenia.. Trends Neurosci.

[15] Durstewitz D, Seamans J (2002). The computational role of dopamine d1 receptors in prefrontal cortex.. Neural Net.

[16] Kischka U, Kammer T, Maier S, Weisbrod M, Thimm M (1996). Dopaminergic modulation of semantic network activation.. Neuropsychologia.

[17] Cools R (2006). Dopaminergic modulation of cognitive function-implications for l-dopa treatment in parkinson's disease.. Neuroscience and Biobehavioral Reviews.

[18] Tivarus M, Hillier A, Schmalbrock P, Beversdorf D (2008). Functional connectivity in an fmri study of semantic and phonological processes and the effect of l-dopa.. Brain Lang.

[19] Demb J, Desmond J, Wagner A, Vaidya A, Glover G (1995). Semantic encoding and retrieval in the left inferior prefrontal cortex: a functional mri study of task difficulty and process specificity.. J Neurosci.

[20] Gold B, Buckner R (2002). Common prefrontal regions coactivate with dissociable posterior regions during controlled semantic and phonological tasks.. Neuron.

[21] Poldrack R, Wagner A, Prull M, Desmond J, Glover G (1999). Functional specialization for semantic and phonological processing in the left inferior prefrontal cortex.. NeuroImage.

[22] Shivde G, Thompson-Schill S (2004). Dissociating semantic and phonological maintenance using fmri.. Cogn Affect Behav Neurosci.

[23] Schmahmann J, Sherman J (1998). The cerebellar cognitive affective syndrome.. Brain.

[24] Beversdorf D, Ratcliffe N, Rhodes C, Reeves A (1997). Pure alexia: clinical-pathologic evidence for a lateralized visual language association cortex.. Clin Neuropathol.

[25] Chalfin B, Cheung D, Muniz J, Silveria L, Finlay B (2007). Scaling of neuron number and volume of the pulvinar complex in new world primates: comparisons with humans, other primates, and mammals.. J Comp Neurol.

[26] Asanuma C, Andersen R, Cowan W (1985). The thalamic relations of the caudal inferior parietal lobule and the lateral prefrontal cortex in monkeys: divergent cortical projections from cell clusters in the medial pulvinar nucleus.. J Comp Neurol.

[27] Trojanowski J, Jacobson S (1976). Areal and laminar distribution of some pulvinar cortical efferents in rhesus monkey.. J Comp Neurol.

[28] Kievit J, Kuypers H (1977). Organization of the thalamo-cortical connexions to the frontal lobe in the rhesus monkey.. Exp Brain Res.

[29] Selemon L, Goldman-Rakic P (1988). Common cortical and subcortical targets of the dorsolateral prefrontal and posterior parietal cortices in the rhesus monkey: evidence for a distributed neural network subserving spatially guided behavior.. J Neurosci.

[30] Markowitsch H (1995). Which brain regions are critically involved in the retrieval of old episodic memory?. Brain Res Rev.

[31] Liddell B, Brown K, Kemp A, Barton M, Das P (2005). A direct brainstem-amygdala-cortical ‘alarm’ system for subliminal signals of fear.. Neuroimage.

[32] Ketteler D, Kastrau F, Vohn R, Huber W (2008). The subcortical role of language processing. high level linguistic features such as ambiguity-resolution and the human brain; an fmri study.. Neuroimage.

[33] Kastner S, Pinsk M (2004). Visual attention as a multilevel selection process.. Cognitive, Affect Behav Neurosci.

[34] Bell A, Sejnowski T (1995). An information-maximization approach to blind separation and blind deconvolution.. Neural Comput.

[35] McKeown M, Makeig S, Brown G, Jung T, Kindemann S (1998). Analysis of fmri data by blind separation into spatial independent component analysis.. Hum Brain Mapp.

[36] Calhoun V, Adali T, Pearlson G, Pekar J (2001). A method for making group inference from functional mri data using independent component analysis.. Hum Brain Mapp.

[37] Beckmann C, Smith S (2004). Probabilistic independent component analysis for functional magnetic resonance imaging.. IEEE Trans Med Imaging.

[38] Beckmann C, DeLuca M, Devlin J, Smith S (2005). Investigations into resting-state connectivity using independent component analysis.. Phil Trans R Soc B.

[39] Eichele T, Calhoun V, Debener S (2009). Mining eeg-fmri using independent component analysis.. Int J Psychophysiol.

[40] Hyvärinen A (1999). Fast and robust fixed-point algorithms for independent component analysis.. IEEE Trans Neural Networks.

[41] Minka T (2000). Atomatic choice of dimensionality for pca.

[42] Svensen M, Kruggel F, Benali H (2002). Ica of fmri group study data.. Neuroimage.

[43] Schmithorst V, Holland S (2004). Comparison of three methods for generating group statistical inferences from independent component analysis of functional magnetic resonance imaging data.. J Magn Reson Imaging.

