# Supplementary material for: Independent Component Analysis of the Effect of L-dopa on fMRI of Language Processing
Source: PLoS One. 2010 Aug 17;5(8):e11933. doi: 10.1371/journal.pone.0011933 (PMC2923146; doi:10.1371/journal.pone.0011933)
Supplement: Text S1 — Correlation threshold calculation. (0.05 MB PDF) [file pone.0011933.s002.pdf]

Text S2. Correlation threshold calculation

By using Fisher's r-to-z transformation, $Z = \frac{1}{2}\ln\frac{1+r}{1-r} \sim N\big(0, \frac{1}{T-3}\big)$, where T is the number of the volume scans in a run. Let $v_i = \frac{2}{\sqrt{T-3}} z_{\alpha_i}$, for a given α_i, i=1,...,Q (number of IC for a subject within one condition). The correlation threshold with significance level α_i satisfies $r_i > \frac{e^{v_i}-1}{e^{v_i}+1}$. In order to adjust multiplicity, we find an error rate α_i for each IC to achieve an overall family error rate α. Assuming independence of these tests, it is known [1] that

$$\Pr\big(\text{At least one Type I error among Q tests}\big) = 1 - \prod_{i=1}^{Q}(1-\alpha_i).$$

Now, using $\alpha_1 = \ldots = \alpha_Q$, the significance level α_i is then given by

$$1 - (1-\alpha_i)^Q < \alpha \rightleftharpoons \alpha_i < 1 - (1-\alpha)^{1/Q}.$$

Thus, e.g., if α=0.05, then for Q=25, the threshold for r is given by $r > 0.33$.

References

1. Hsu J (1996) Multiple comparisons: theory and methods. Chapman & Hall. 11 p.
