# Supplementary material for: Independent Component Analysis of the Effect of L-dopa on fMRI of Language Processing
Source: PLoS One. 2010 Aug 17;5(8):e11933. doi: 10.1371/journal.pone.0011933 (PMC2923146; doi:10.1371/journal.pone.0011933)
Supplement: Text S2 — Assumptions for the test statistics in the fexible factorial model. (0.11 MB PDF) [file pone.0011933.s003.pdf]

Text S3. Assumptions for the test statistics in the flexible factorial model

Let Y_{ijk} (i=A,B; j=1,2; k=1,...,16) denote the observed values of the summary map under task (i) and drug (j) of the kth subject, where letter A (or B) is for the phonological (or semantic) task and the number 1 (or 2) is for L-dopa (or placebo) condition. The design matrix for the flexible factorial model is provided in Figure a).

Spatial distribution of a source estimated by PICA is then fitted by a mixture of three Gaussian distributions including the standard Gaussian distribution [36]. The standard Gaussian distribution is the density for background noise whereas the other two Gaussian distributions are the densities for negative (or deactivated) or positive (or activated) source values, respectively. In this study, voxel-wise significance testing for each task (i) and drug (j) condition is based on the t-test using the sample mean of the summary values Y_{ijk}, k=1,...,16, across subjects, and pooled variance across four conditions. The degrees of freedom for this t-test is equal to 60 (=15x4). The validity of the threshold, $t^*(1-\alpha, 60)$, used in the analysis for voxel-wise significance, was examined through a simulation of the proposed summarizing procedure (1) and (2) in the Materials and Methods section. In this simulation, random samples were generated from background Gaussian distribution of each task-related IC for each individual within each task and drug condition. Distribution of t-ratios for each task and drug was then compared to the t-distribution with 60 d.f.. In Figure b), the distribution of simulated t-ratios for the phonological task with placebo is plotted along with the density of t-distribution with 60 d.f. (dashed line). This plot indicates that the t-distribution with 60 d.f. fits well to the distribution of t-ratios with the summary values based on the random samples generated from background Gaussian distribution. The distribution of observed t-ratios for the phonological task with placebo is provided in Figure c). The distribution of simulated t-ratios is compared to the distribution of the observed t-ratios in Figure d).

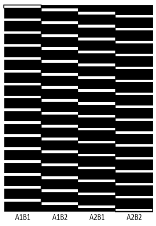

a) Design matrix for SPM

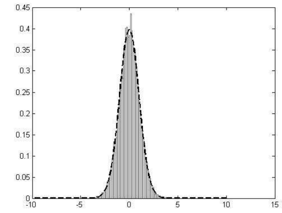

b) Simulated t−ratio for the cell (A, 2) under the null hypothesis

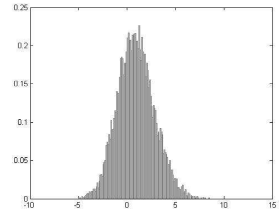

c) Observed t−ratio for the cell (A, 2)

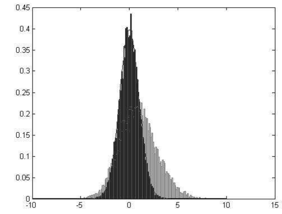

d) Simulated null and observed t−ratios
